# Construction of a consensus linkage map for red clover (*Trifolium pratense *L.)

**DOI:** 10.1186/1471-2229-9-57

**Published:** 2009-05-14

**Authors:** Sachiko Isobe, Roland Kölliker, Hiroshi Hisano, Shigemi Sasamoto, Tshyuko Wada, Irina Klimenko, Kenji Okumura, Satoshi Tabata

**Affiliations:** 1 Kazusa DNA Research Institute, Kazusa-Kamatari 2-6-7, Kisarazu, Chiba, 292-0818, Japan; 2 Agroscope Reckenholz-Tänikon Research Station ART, Reckenholzstr. 191, 8046 Zurich, Switzerland; 3 All-Russian Williams Fodder Crop Research Institute, 141055 Lugovaya, Moscow Region, Russia; 4 National Agricultural Research Institute for Hokkaido Region, Hitsujigaoka 1, Toyohira, Sapporo, 062-8555, Japan; 5 Samuel Robert Noble Foundation. 2510 Sam Noble Pky. Ardmore, OK, 73401, USA

## Abstract

**Background:**

Red clover (*Trifolium pratense *L.) is a major forage legume that has a strong self-incompatibility system and exhibits high genetic diversity within populations. For several crop species, integrated consensus linkage maps that combine information from multiple mapping populations have been developed. For red clover, three genetic linkage maps have been published, but the information in these existing maps has not been integrated.

**Results:**

A consensus linkage map was constructed using six mapping populations originating from eight parental accessions. Three of the six mapping populations were established for this study. The integrated red clover map was composed of 1804 loci, including 1414 microsatellite loci, 181 amplified fragment length polymorphism (AFLP) loci and 204 restriction fragment length polymorphism (RFLP) loci, in seven linkage groups. The average distance between loci and the total length of the consensus map were 0.46 cM and 836.6 cM, respectively. The locus order on the consensus map correlated highly with that of accession-specific maps. Segregation distortion was observed across linkage groups. We investigated genome-wide allele frequency in 1144 red clover individuals using 462 microsatellite loci randomly chosen from the consensus map. The average number of alleles and polymorphism information content (PIC) were 9.17 and 0.69, respectively.

**Conclusion:**

A consensus genetic linkage map for red clover was constructed for the first time based on six mapping populations. The locus order on the consensus map was highly conserved among linkage maps and was sufficiently reliable for use as a reference for genetic analysis of random red clover germplasms.

## Background

Red clover is widely cultivated in most temperate regions of the world as a forage legume and as green manure. Red clover is an outcrossing species, with a diploid genome (2n = 2X = 14) of approximately 440 Mb [1]. Currently, three genetic linkage maps have been published for red clover. The first linkage map, containing 158 loci over a total length of 535.7 cM, was constructed in 2003 by Isobe et al. [2] using RFLP markers derived from red clover cDNAs. A high-density linkage map containing 1434 loci over a total length of 868.7 cM was developed in 2005 by Sato et al. using primarily microsatellite markers [1]. In 2006, Herrmann et al. reported an AFLP and microsatellite-based map containing 258 loci over a total length of 444.2 cM [3].

Because red clover has a strong gametophytic incompatibility system, the present varieties have developed mainly by mass selection, recurrent selection and natural selection [4,5]. The use of breeding methods that improve specific traits while maintaining genetic diversity in a variety of red clover has resulted in abundant intra-population genetic diversity [6,7]. This high level of genetic diversity in red clover is also evident in polymorphism analyses using RFLP, AFLP and microsatellite markers 1, 2, 3, 8, 9, 10. While it is highly probable that the DNA markers of the three currently available red clover linkage maps are transferable across random germplasms, it is also likely that a locus position on a random red clover germplasm will be shifted from its original position in the mapping population due to segregation distortion or chromosome rearrangement. In previous linkage map studies, subsets of RFLP and microsatellite markers were used to determine the correspondence between linkage groups, but data related to the stability of locus positions in each linkage group was not reported.

For several crop species, such as maize [11,12], soybean [13,14], barley 15, 16, 17, grapevine 18, 19, 20 and lettuce [21], integrated consensus linkage maps that combine information from multiple mapping populations have been developed. These maps are generally constructed with the aim of determining the relative position of transferable markers, increasing the number of available DNA markers, obtaining saturated maps and comparing the locations of quantitative trait loci (QTL) and candidate genes of interest across germplasms. Similarly, the construction of a consensus linkage map for red clover should enable us to determine the stability of locus positions across random red clover germplasms, as well as increase the number of loci in the linkage map.

In addition to the construction of informative linkage maps, genome-wide polymorphism analysis has been a recent focus in QTL detection and genomics-based, marker-assisted breeding in an attempt to harness the genomic diversity of a targeted species [22]. In red clover, Herrmann et al. (2006) identified 38 candidate QTL relating to seed yield components using a F_1 _mapping population [3]. However, there have been no reports identifying QTL based on the diverse genetic variation in red clover germplasms. Investigation of genome-wide polymorphisms, along with the construction of consensus map positions of each marker, is integral to our ability to carry out genetic analyses of red clover, a species that exhibits a high level of genetic diversity.

In the current study, we developed a consensus linkage map for red clover that integrates DNA markers from three previously reported maps with segregation data from six mapping populations, including three newly generated populations. By comparing the locus order on the consensus map and each accession-specific map, we were able to estimate the robustness and saturability of the consensus linkage map. In addition, genome-wide allele frequencies in 1144 red clover individuals, derived from 48 varieties/lines from different regions of the world and parents of mapping populations, were estimated using 462 microsatellite loci randomly chosen from the consensus map.

## Results

### Construction of a consensus genetic linkage map

A total of 1770 markers, including 1391 microsatellite, 251 AFLP, 121 RFLP and 6 random amplified polymorphic DNAs (RAPD) markers, and 1 sequence tagged site (STS) marker, were used for the construction of a linkage map. A total of 4043 genotypes were generated from 12 mapping populations representing 8 red clover parental accessions (Table 1). The largest data sets were from the parental accession HR, followed by R130, and were derived from HR × R130 crosses. The polymorphism ratio of 234 bridging microsatellite markers, which were previously developed for HR × R130 or pC × pV crosses, ranged from 35.0% to 70.0% in the other parental accessions.

**Table 1:** Description of the mapping population, number of genotyped loci and polymorphic ratio of the bridging markers.

			Number of segregation data sets						
				
Accession name	Mapping population	Number of mapping progenies	Microsatellite	AFLP	RFLP	RAPD	STS	Total	Polymorphic ratio of the bridging markers (%)^a)^
HR	HR × R130	188	1004	0	109	0	0	1113	-
	NS10 × HR	94	158	0	0	0	0	158	-

NS10	NS10 × HR	94	156	0	0	0	0	156	65.8
	NS10 × H17L	94	121	0	0	0	0	121	51.1

H17L	NS10 × H17L	94	166	0	0	0	0	166	70.0
	H17L × R130	94	122	0	0	0	0	122	51.5

R130	H17L × R130	94	126	0	0	0	0	126	-
	HR × R130	188	792	0	109	0	100	1001	-

272	272 × WF1680	94	123	0	197	5	0	325	51.9
WF1680	272 × WF1680	94	83	0	147	3	1	234	35.0

pC	pC × pV	254	143	134	0	0	0	277	-
pV	pC × pV	254	124	120	0	0	0	244	-

Total			3118	254	562	8	101	4043	

^a)^A total of 234 Bridging microsatellite markers were selected from the HR × R130 and pC × pV maps.

The integrated red clover map was composed of 1804 loci (1414 microsatellite loci, 181 AFLP loci, 204 RFLP loci, 2 RAPD loci, and 1 STS locus) in seven linkage groups (Table 2). A total of 260 loci detected by 234 bridging microsatellite markers allowed the integration of the 12 individual segregation data sets into a consensus linkage map. Marker information, including position on the consensus map, marker type and bridging marker are listed in Additional file 1: Table S1. The total length of the consensus map was 836.6 cM, 648.0 cM of which were covered by the bridging microsatellite markers (Table 2). The length of the linkage groups ranged from 102.2 cM (LG7) to 138.8 cM (LG2), and 64.70% (LG5) to 90.0% (LG2) of each linkage group was covered by bridging markers. The average distance between loci was 0.46 cM, and ranged from 0.39 cM (LG7) to 0.59 cM (LG5). The largest gap between two loci was approximately 13.6 cM, between C1984 (125.1 cM) and TPSSR17 (138.8 cM) in LG2, and between RCS2987 (10.4 cM) and RCS1155 (24.0 cM) in LG5. Locus density tended to be lower in the distal regions of each linkage group (See Additional file 2: Fig. S1).

**Table 2:** Description of the consensus linkage map.

	Length (cM)			Number of Loci							
				
	Consensus map	Bridging marker ^a)^		Micro satellite	AFLP	RFLP	STS·RAPD	Total ^b)^		Average distance between two loci ^c)^	PIC ^d)^
Total	836.6	648.0		1414	181	204	3	1804	(260)	0.46	0.69
LG1	128.5	102.1	(0.0–102.1)	182	30	11	1	224	(38)	0.57 (0.0–9.0)	0.68
LG2	138.8	124.9	(13.9–138.8)	266	35	38	-	339	(40)	0.41 (0.0–13.6)	0.71
LG3	119.3	86.9	(22.1–109.0)	226	22	47		295	(35)	0.40 (0.0–7.1)	0.67
LG4	117.9	102.2	(3.8–106.0)	210	31	33	-	274	(39)	0.43 (0.0–8.3)	0.69
LG5	120.7	78.1	(42.6–120.7)	152	27	26	-	205	(35)	0.59 (0.0–13.6)	0.69
LG6	109.2	86.5	(16.0–102.5)	163	17	19	1	200	(37)	0.55 (0.0–7.5)	0.71
LG7	102.2	71.4	(26.4–97.8)	215	19	30	1	265	(36)	0.39 (0.0–7.1)	0.68

^a) ^Map length covered with bridging markers. Parenthesis show both ends of the marker positions

^b) ^Parenthesis show the number of loci detected by the bridge microsatellite markers.

^c) ^Parenthesis show the range of marker density.

^d) ^Markers those generated multiple loci were excluded from the calculation.

On the consensus map, 47 microsatellite markers (including 27 bridging markers; 3.4% of the total) and 48 RFLP markers (38.7% of the total) generated multiple loci (See Additional file 1: Table S1). The average number of loci per microsatellite and RFLP marker was 2.0 and 2.1, respectively. The range of loci per microsatellite marker (2–3) was smaller than the range of loci per RFLP marker (2–11). Each locus detected by identical microsatellite markers mapped to a multi-linkage group, while multiple loci detected by identical RFLP markers did not always map to multi-linkage groups.

### Comparison of accession-specific linkage maps and the consensus map

The total number of loci on the accession specific maps ranged from 191 (H17L) to 997 (HR) (Table 3). The ratio of mapped to analyzed loci differed depending on the population. NS10 and H17L exhibited higher ratios (97.9–100%), while 272 and WF1680 exhibited lower ratios (54.3–65.5%). The length of each accession-specific map differed, ranging from 504.6 cM to 829.0 cM, but none of the accession maps exceeded the length of the consensus map. The segregation distortion ratio of the tested markers and mapped loci on the accession-specific maps ranged from 5.8% (H17L) to 45.0% (272), and from 5.6% (H17L) to 22.7% (R130), respectively (Table 4). The parents of the 272 × WF1680 cross exhibited the two highest segregation distortion ratios for tested markers, while R130 exhibited the highest segregation distortion ratio for mapped loci. H17L exhibited the lowest segregation distortion ratio for both tested markers and mapped loci. Segregation distortion was randomly observed across linkage groups (See Additional file 1: Figure S1). However, the segregation distortion ratio of each linkage group varied, and the most distorted linkage group differed among the accessions (Table 4). For example, LG7 exhibited the highest segregation distortion ratio among all linkage groups on pC-specific (71.0%) and WF1680-specific (68.4%) maps, whereas it exhibited the lowest segregation distortion ratio on the H17L-specific map (0%).

**Table 3:** Comparison between the accession-specific maps and the consensus map

Accession name	Number of genotype data set	Number of analyzed loci	Number of mapped loci ^a)^		Total length of the map (cM) ^b)^		Average distance between two loci (cM)
HR	1271	1113	997	(89.6)	813.6	(97.2)	0.8
R130	1127	1001	810	(80.9)	748.6	(89.5)	0.9

pC	277	277	228	(82.3)	504.6	(60.3)	2.2
pV	244	240	201	(83.8)	531.6	(63.5)	2.6

272	325	325	213	(65.5)	829.0	(99.1)	3.9
WF1680	234	234	127	(54.3)	571.9	(68.4)	4.5

NS10	277	196	180	(91.8)	514.2	(61.5)	2.9
H17L	288	195	191	(97.9)	560.0	(66.9)	2.9

Consensus Map	4043	1899	1804	(95.0)	836.6		0.46

^a) ^Parenthesis show the ratio to the number of tested loci.

^b) ^Parenthesis show the ratio to the length of the consensus map.

**Table 4:** Segregation distortion ratio (%) of the tested markers and the mapped loci on the accession specific maps. ^a)^

		Mapped loci							
		
Accession name	Tested markers	LG1	LG2	LG3	LG4	LG5	LG6	LG7	Total
HR	19.2	39.1	28.4	15.5	9.2	5.0	18.3	9.0	18.9
R130	26.1	11.1	63.0	15.8	22.2	5.2	9.3	7.3	22.7

pC	24.4	0.0	0.0	9.4	0.0	0.0	4.8	71.0	1.4
pV	28.4	12.5	7.0	3.3	0.0	0.0	72.7	22.7	14.6

272	45.0	60.0	12.1	5.1	11.4	12.5	20.0	40.7	20.8
WF1680	44.5	0.0	7.7	19.2	28.6	20.0	6.7	68.4	20.6

NS10	20.5	18.6	37.2	6.3	10.3	3.7	8.1	53.8	19.4
H17L	5.8	2.1	4.4	2.9	5.0	13.2	10.5	0.0	5.6

a) A significant at P < 0.05.

**Figure 1:**
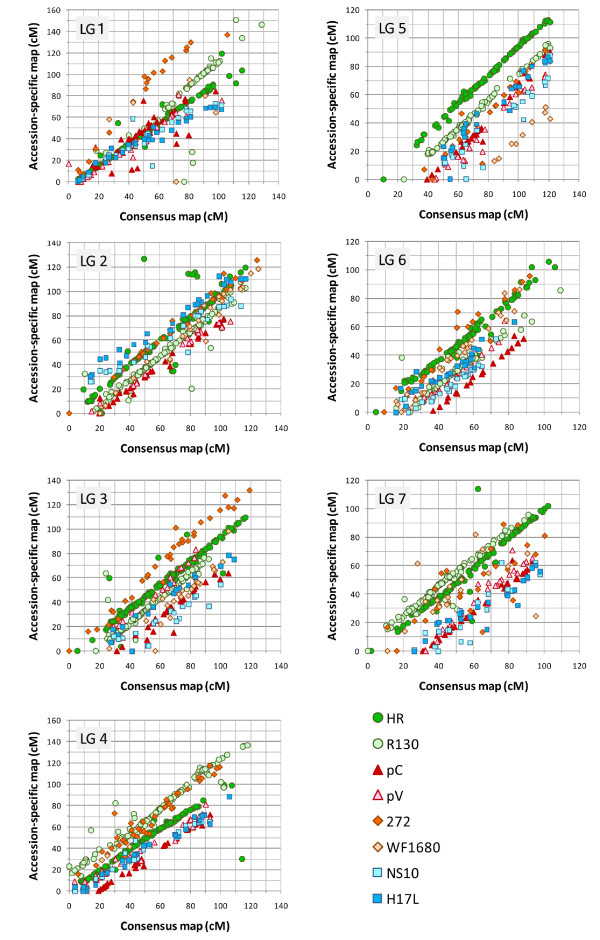
**Comparison of loci positions in the consensus map and accession specific maps**. HR, R130, pC, pV, 272, WF1680, NS10 and H17L are indicated by green circles, light-green circles, red triangles, pink triangles, orange diamonds, light-orange diamonds, light-blue squares and blue squares, respectively.

Locus order was well conserved between the consensus map and accession-specific maps for all linkage groups (Fig. 1), with the exception of loci in LG1 of the WF1680 map, which did not correlate significantly (P < 0.05) with the consensus map (Table 5). LG1 and LG7 exhibited a slightly scrambled locus order between the consensus map and the accession-specific maps. The loci on 110–120 cM of LG2 in the HR-specific map were not located at the corresponding positions of the consensus map (Fig. 1). The locus density in the distal regions of the accession-specific maps tended to be lower than in the proximal regions, as was observed for the consensus map.

**Table 5:** Correlation coefficient for marker positions between each accession specific map and the consensus map.

	HR	R130	pC	pV	272	WF1680	NS10	H17L
LG1	0.99**	0.81**	0.85**	0.96**	0.97**	0.20	0.92**	0.98**
LG2	0.93**	0.96**	0.99**	0.99**	0.99**	0.98**	0.96**	0.99**
LG3	0.98**	0.92**	0.96**	0.99**	0.99**	0.91**	0.94**	0.95**
LG4	0.95**	0.98**	1.00**	0.98**	0.96**	0.96**	0.99**	0.99**
LG5	1.00**	1.00**	0.99**	0.99**	0.95**	0.97**	0.94**	0.97**
LG6	0.99**	0.95**	0.99**	0.97**	0.96**	0.98**	0.93**	0.94**
LG7	0.97**	0.94**	1.00**	0.95**	0.77**	0.56*	0.92**	0.92**

** and * indicates P < 0.01 and P < 0.05, respectively.

### Genome-wide allele frequency in red clover germplasms

The genome-wide allele frequencies of 462 microsatellite loci randomly mapped onto the consensus map were estimated based on the number of alleles and PIC for 1144 red clover individuals originating from 48 varieties and HR, R130, NS10 and H17L. The list of loci is presented in Additional file 1: Table S1. Prior to estimating allele frequency, population structure was estimated using Structure ver.2.2 software. Statistics were computed for K = 2 to 5, where K represents the number of subpopulations, and the maximum P value representing the allele-frequency divergence among subpopulations was distributed from 0.0035 (K = 2) to 0.0343 (K = 5). The results were indicative of the absence of population structure in the 1144 red clover individuals.

**Figure 2:**
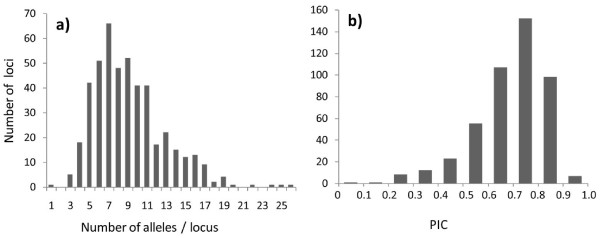
**Allele frequency in 1144 red clover individuals**. (a) Distribution of the number of alleles per locus; (b) Distribution of PIC.

The number of alleles generated for each locus ranged from 1 to 26, with an average value of 9.17, and PIC ranged from 0.09 to 0.92, with an average value of 0.69 (Fig. 2). The average PIC value for each linkage group in the consensus map ranged from 0.67 to 0.71 (Table 2). PIC values varied among linkage groups (See Additional file 2: Fig S1).

## Discussion

There are currently no generally accepted standards for defining or naming integrated linkage maps. As a result, integrated maps are alternately referred to as consensus, composite, pooled, comprehensive, reference or integrated maps, depending on the integration procedure and characteristics, as well as the reason for generating the map [23]. In the current study, we constructed an integrated linkage map for red clover using a regression mapping algorithm of JoinMap ver.4, which is based on mean recombination frequencies, and combined multiple segregation data sets [24]. The order of the mapped loci was generally well conserved between the integrated map and the accession-specific maps, which indicated that the positions of the loci on the present integrated map can be regarded as the "consensus" positions. For this reason, we have termed our integrated map a "consensus map".

The average distance between loci and total length of the consensus map were 0.46 cM and 836.6 cM, respectively. Our consensus map had a higher locus density and was slightly shorter than a previously reported saturated linkage map (HR × R130 map), in which the average distance between loci and total length were 0.61 cM and 868.7 cM, respectively [1]. The lengths of the HR-specific and R130-specific maps reconstructed in this study were 813.6 cM and 748.6 cM, respectively, and were shorter in length than previously reported maps. Based on these results, we conclude that the red clover consensus map developed in the current study is saturated, and that the mapping algorithm used to generate the map likely has a slight influence on the total length. However, there were still several gaps in the distal regions of the linkage groups, as observed by visual inspection. The results of genome-wide PIC assessment suggested that there are no clear differences in allelic polymorphisms across the genomes. Therefore, the reduced locus density in distal regions may be due to other factors, such as the structural features of the chromosomes, or alternatively, statistical issues. One of the largest gaps in the map was 13.6 cM (between RCS2987 and RCS1155), in LG5. LG5 corresponds to chromosome 1, which has been shown by fluorescence *in situ *hybridization (FISH) to include large regions on the short arm that hybridize with 28S rDNA [1]. The presence of this large hybridization region might prevent or hamper the identification of polymorphic markers in this region, leading to an apparent lower locus density in the upper region of LG5.

The quality of the genotyping data is a critical element in linkage analysis [25]. A three percent error rate in genotyping can double the genetic map length [26]. In the current study, the total length of the consensus map was 836.6 cM, and bridging markers covered 648.0 cM of the linkage map, which suggests that the distal regions of the linkage groups were not well covered by bridging markers. Thus, reduced multiple segregation data or a genotyping error might be more factors contributing to the lower locus density in the distal regions of the linkage groups.

Segregation distortion was observed across the linkage groups. The distortion ratios of the tested markers, as well as for mapped loci, were different among the red clover accessions. For the tested markers, WF1680 and 272 exhibited the highest distortion ratio, nearly 7.5 times higher than that of H17L, which exhibited the lowest distortion ratio. However, many of the skewed loci in WF1680 and 272 were excluded during the mapping procedure, and as a result, R130 exhibited the highest segregation distortion ratio for mapped loci. The segregation distortion ratios of each linkage group varied widely in each accession, and interestingly, the most skewed linkage group differed according to accession-specific map. These results suggest that segregation distortion in red clover can occur anywhere in the genome, in an accession-specific manner.

Locus order was generally well conserved; however, the robustness of the locus order differed slightly depending on the linkage group and the accession-specific linkage map. The weakest correlations of locus order between the consensus map and an accession-specific map were for LG1 and LG7 in the WF1680-specific map. WF1680 exhibited the lowest polymorphic ratio of bridging markers, which might be due to the close genetic distance between the two haplotype genomes in WF1680. The close genetic distance between the two haplotype genomes might also explain the fact that WF1680 also had the second highest segregation distortion ratio for tested markers and the lowest locus density, both of which would cause unstable locus order.

Hayashi et al. (2001) reported that differences in locus order on a linkage map represent chromosomal rearrangements in *Lotus japonicus *[27]. In the current study, the loci in the 110–120 cM region of LG2 in the HR-specific map were not located in the corresponding position on the consensus map. These results suggest the possibility of a chromosomal rearrangement in this region. However, the overall conservation of locus order indicates that chromosomal rearrangements have not occurred frequently in red clover.

Microsatellite and RFLP markers occasionally detected multiple loci. It is possible that these markers detected paralogous regions that do not always give rise to polymorphisms in each parental combination. RFLP markers generated multiple loci more often than microsatellite markers, which suggests that microsatellite markers are more suitable than RFLP markers as consensus markers. However, the larger percentage of bridging microsatellite markers (12.1%) that detected multiple loci as compared to total microsatellite markers (3.4%) emphasizes that care must be taken with respect to multiple loci when carrying out marker analysis using various unrelated accessions in red clover.

The average number of alleles per microsatellite locus and PIC in 1144 red clover individuals was 9.17 and 0.69, respectively. This is an intermediate level of polymorphism relative to the results of Sato et al. (average allele number and PIC, 6.5 and 0.60, respectively) and Dias et al. (average allele number and PIC, 11.1 and 0.86, respectively) [1,10]. Because the number of loci and red clover individuals that were tested in the current study were extremely large compared to these two previous reports, the results of the current study likely represent values that are more typical for red clover germplasms.

Using the genome-wide allele frequency data of 1144 red clover individuals and 462 microsatellite loci, we carried out a preliminary estimate of the extent of linkage disequilibrium (LD, D') using the GGT 2.0 program [28]. There was no significant correlation between D' and distance between two loci (See Additional file 3: Fig S2). This result suggests that the extent of LD in red clover is low. For a highly heterozygous species, LD mapping is a more effective approach to QTL detection than interval mapping, as it captures a wider spectrum of genetic diversity. However, LD mapping is more difficult in a heterozygous species than in a homozygous species, because the extent of LD is likely to be small, and, therefore, more markers are required to detect significant associations between marker genotypes and specific traits. The dense consensus linkage map developed in this study will accelerate LD mapping in red clover, as well as QTL detection by interval mapping.

## Conclusion

We have constructed the first consensus linkage map for red clover. The locus order of the present consensus map is highly consistent, and is sufficiently reliable for use as a reference for the genetic analysis of random red clover germplasms. The consensus map and genome-wide polymorphic information provided by the current study will facilitate further genetic advances in the molecular breeding of red clover in the near future.

## Methods

### Construction of a consensus linkage map

#### Plant material

A consensus linkage map was constructed using six mapping populations originating from eight parental accessions (Table 1). Three of the six populations were previously described. The 272 × WF1680 population was a BC_1_F_1 _population of 94 individuals in which the parent '272' was a single F_1 _plant from a cross between '1588', a wild specimen collected in the Arhangelsk region of Russia, and 'WF1680', which originated from a central Russian variety [2]. HR × R130 was a one-way pseudo-testcross mapping population of 188 individuals in which the female parent, 'HR', originated from the Japanese variety 'Hokuseki', and the male parent 'R130' was a progeny of 272 × WF1680 [1]. pC × pV was a two-way pseudo-testcross population of 254 individuals created with the 'pC' genotype from the Swiss Mattenklee variety 'Corvus' and the 'pV' genotype from the Belgian cultivar 'Violetta' [3].

The other three populations, NS10 × HR, NS10 × H17L and H17L × R130, were developed for this study. 'NS10' was a genotype that originated from the Japanese variety 'Natshyu'; 'H17L' was derived from a breeding line of the National Agricultural Research Center for Hokkaido Region (Japan) and originated from a cross between Finnish varieties, 'Nolac' and 'Hankkijan-Venla', and the Canadian variety 'Tanila'. Each population was a one-way pseudo-testcross of 94 individuals.

#### Marker Analysis

Segregation data sets of RFLP, AFLP and microsatellite markers mapped on previous red clover maps were used for the construction of the consensus map (Table 1) 1, 2, 3. Markers designated with a single 'C' and a number indicate RFLP markers, while 'C_PK_' and 'V_PK_' followed by a number represent AFLP markers. 'TPSSR' and 'RCS' designate microsatellite markers. 'TPSSR' markers were obtained from simple sequence repeat (SSR)-enriched genomic libraries [28], and 'RCS' markers were primarily developed using expressed sequence tags (ESTs). All primer information for the microsatellite markers is available in Kölliker et al. [29], or at the Clover GARDEN website . The segregation data sets for RFLP markers were derived from the 272 × WF1680 and HR × R130 mapping populations, while the segregation data for AFLP markers was derived from the pC × pV mapping population. The segregation data of two RAPD markers ('OPB' markers) and one STS marker ('SICAS'), which were not previously reported, were obtained using the HR × R130 mapping population. Operon^® ^10 mer primer kits B and C (Operon Technologies, USA) were used for RAPD marker development. The SAICAS primer sequences were as follows: 5'-TAGAGGAGTTGTGGACAAGA and 5'-TAGATACATGAGGTGATAAGA.

A total of 234 microsatellite markers, including 224 RCS and 15 TPSSR markers, were tested in the polymorphism analysis using all mapping populations to generate bridging markers for the consensus map. PCR was performed in a reaction volume of 5 μl containing 0.5 ng of red clover genomic DNA, 0.2 mM dNTPs, 3 mM MgCl_2_, 0.4 μM each of the primer pairs and 0.2 U Takara rTaq with 1× PCR buffer (Takara Bio Inc., Japan) or 0.04 U BIOTAQ™ DNA Polymerase with 1× NH_4 _Buffer (BIOLINE, UK). For amplification, we used the modified 'touchdown PCR' program [30] of Sato et al. (2005) [1]. Amplified products were resolved by 10% acrylamide gel electrophoresis.

#### Linkage analysis

A combination of the color map method and the JoinMap program ver.4 was used to analyze the segregation data sets obtained from each mapping population [28,31]. First, the scored markers were roughly classified into seven linkage groups using the color map method. Next, the robustness of the data sets for each linkage group was confirmed by the grouping module of JoinMap using an logarithm of odds (LOD) threshold of 2.0. For the construction of a consensus linkage map, allele data sets related to the same linkage groups with at least two loci in common were integrated into one data set by applying the 'combine groups for map integration' module. The locus order was calculated using a regression mapping module of JoinMap and the following parameters: Kosambi's mapping function, LOD ≥ 2.0, REC frequency ≤ 0.4, goodness-of-fit Jump threshold for removal loci = 5.0, number of added loci after which to perform a ripple = 1, and third round = Yes.

A total of eight individual maps were developed for HR, R130, NS10, H17L, 272, WF1680, pC, and pV. Because two data sets each were generated for HR, R130, NS10 and H17L, the two data sets were integrated into one data set by the 'combine groups for Map integration' module, and then ordered by the regression mapping module of JoinMap. The data sets of 272, WF1680, pC and pV were directly applied to the regression mapping module to order the locus. Parameters used for the mapping module of the individual maps were same as the consensus map.

### Genome-wide allele frequency

#### Plant material and marker analysis

A total of 1144 individuals were used for polymorphism analysis with microsatellite loci, including the four mapping parents HR, R130, NS10 and H17L. The other 1140 individuals were selected from 48 varieties bred in different regions of the world (See Additional file 4 Table S2). The number of individuals tested per variety ranged from 9 to 40. A total of 462 'RCS' markers randomly mapped and generated single locus on the were used for polymorphism analysis (See Additional file 1: Table S1). PCR and polymorphic band detection were performed under the same conditions as described for the construction of the consensus map.

#### Data analysis

Allele detection and genotype code typing were performed using the BioNumerics program, ver.4.6 (Applied Maths BVBA, Sint-Martens-Latem, Belgium). The presence or absence of amplification and the number of different-sized fragments, which was taken as the number of alleles, were recorded. Loci for which there was no amplification were designated as null alleles. Structure ver2.2 software was employed to determine the number of alleles, the heterozygous/homozygous ratio of single amplification fragments, and identify the population structure [32,33] with the following parameters: length of burning period = 10,000; number of MCMC population in the burning period = 10,000. PIC was calculated using the following equation:



where P_ij _is the frequency of the *jt*h allele for the *i*th locus.

## Authors' contributions

SI conceived the study, participated in its design, performed the data analysis, and coordinated the work on the manuscript. RK and IK provided the genotype data and helped to draft the manuscript. HH, SS and TY participated in obtaining the genotyping data. KO carried out the construction of the mapping population. ST participated in obtaining the genotyping data and helped to draft the manuscript.

## Supplementary Material

Additional file 1 **Consensus map position and marker type for each locus**. The data provided the description of consensus map. Click here for file

Additional file 2 **Consensus linkage map for red clover, distribution of PIC and segregation distortion ratio according to linkage group**. The figure shows a consensus linkage map for red clover, distribution of PIC and segregation distortion ratio according to linkage group. The middle bar in each linkage group indicates the consensus linkage map. Blue and red dots show the distribution of PIC and distortion ratio, respectively. The segregation distortion ratio of each locus was calculated using the following formula: (Number of distorted individual segregation data sets) × 100/number of polymorphic individual segregation data sets. Click here for file

Additional file 3 **Distribution of LD between microsatellite markers in each linkage group in relation to genetic distance**. The figure shows distribution of LD between microsatellite markers in each linkage group in relation to genetic distance (cM). Red, orange, yellow, green, aqua, blue and purple dots indicate marker pairs of LG1, LG2, LG3, LG4, LG5, LG6 and LG7, respectively. LD (D') was measured using the GGT 2.0 program based on the genome-wide polymorphic data of 1144 red clover individuals × 462 microsatellite markers. Click here for file

Additional file 4 **List of plant materials used for genome-wide polymorphic analysis**. The data provided the plant materials used for genome-wide polymorphic analysis. Click here for file
