# Supplementary figures and images for: Construction of a consensus linkage map for red clover (Trifolium pratense L.)
Source: BMC Plant Biol. 2009 May 14;9:57. doi: 10.1186/1471-2229-9-57 (PMC2695442; doi:10.1186/1471-2229-9-57)

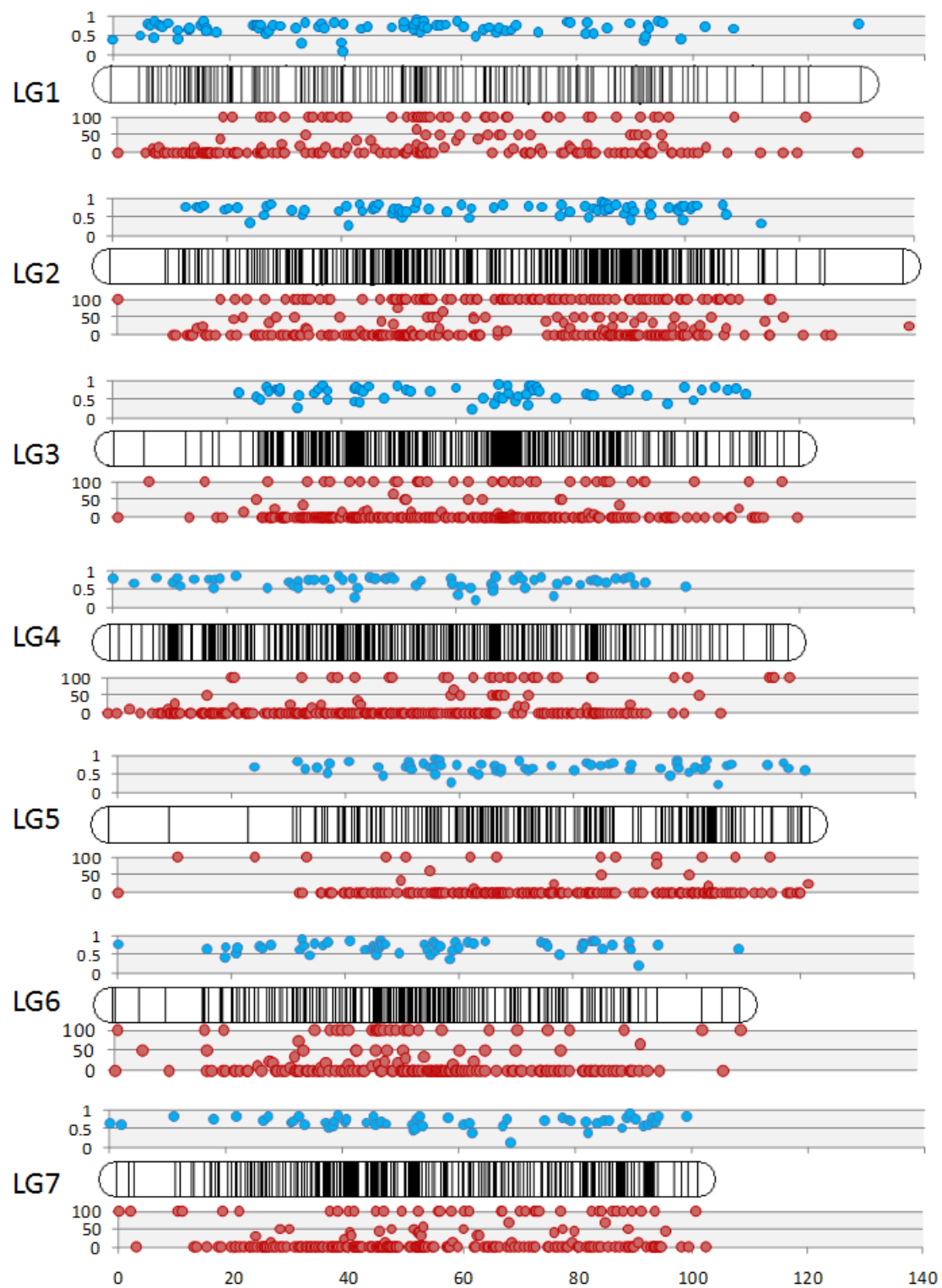

Supplement: Additional file 2 — Consensus linkage map for red clover, distribution of PIC and segregation distortion ratio according to linkage group. The figure shows a consensus linkage map for red clover, distribution of PIC and segregation distortion ratio according to linkage group. The middle bar in each linkage group indicates the consensus linkage map. Blue and red dots show the distribution of PIC and distortion ratio, respectively. The segregation distortion ratio of each locus was calculated using the following formula: (Number of distorted individual segregation data sets) × 100/number of polymorphic individual segregation data sets. [file 1471-2229-9-57-S2.pdf]

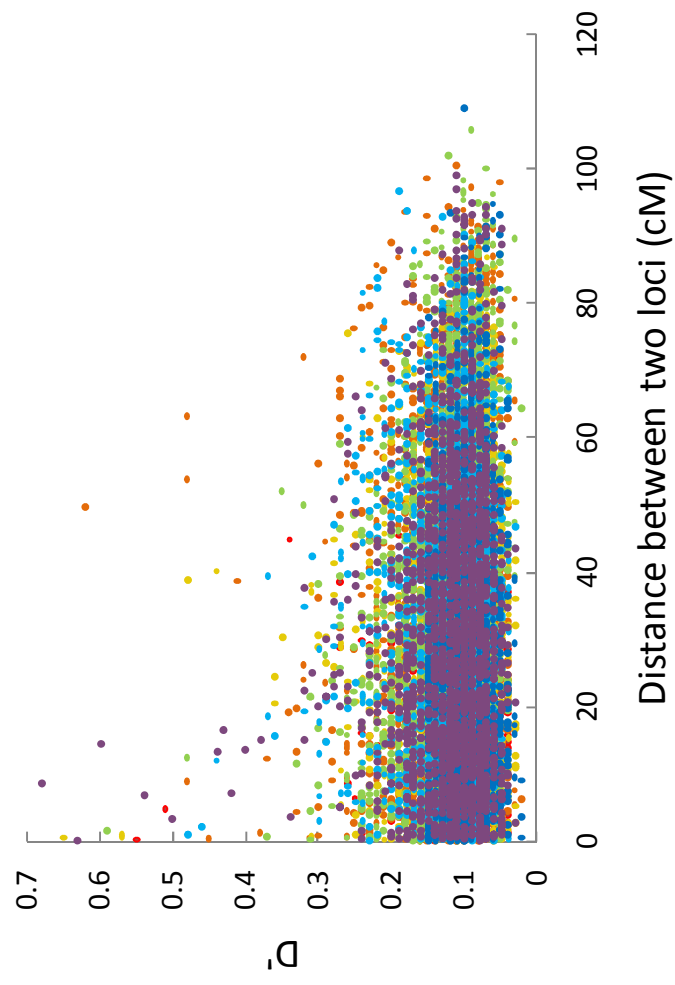

Supplement: Additional file 3 — Distribution of LD between microsatellite markers in each linkage group in relation to genetic distance. The figure shows distribution of LD between microsatellite markers in each linkage group in relation to genetic distance (cM). Red, orange, yellow, green, aqua, blue and purple dots indicate marker pairs of LG1, LG2, LG3, LG4, LG5, LG6 and LG7, respectively. LD (D') was measured using the GGT 2.0 program based on the genome-wide polymorphic data of 1144 red clover individuals × 462 microsatellite markers. [file 1471-2229-9-57-S3.pdf]
